# HDAC and HAT inhibitors differently affect analgesia mediated by group II metabotropic glutamate receptors

**DOI:** 10.1186/1744-8069-10-68

**Published:** 2014-11-18

**Authors:** Magda Zammataro, Maria Angela Sortino, Carmela Parenti, Robert W Gereau, Santina Chiechio

**Affiliations:** Department of Drug Sciences, Section of Pharmacology and Toxicology, University of Catania, 95125 Catania, Italy; Department of Biomedical and Biotechnological Sciences Section of Pharmacology, University of Catania, 95125 Catania, Italy; Washington University Pain Center and Department of Anesthesiology Washington University School of Medicine, St. Louis, MO USA

**Keywords:** Inflammatory pain, mGlu2, Curcumin, Epigenetic modulation, HDAC, HAT

## Abstract

**Background:**

Histone deacetylases (HDACs) and histone acetyltransferases (HATs) are key players in epigenetic regulation of gene expression. Analgesic activity by HDAC inhibitors has been reported in different pain models including inflammatory and neuropathic pain. These drugs interfere with gene expression through different mechanisms including chromatin remodeling and/or activation of transcription factors. Among other targets, HDAC inhibitors regulate metabotropic glutamate receptors type 2 (mGlu2) expression in central and peripheral central nervous system. However whether inhibition of HAT activity also regulates mGlu2 expression has not been reported.

**Findings:**

Here we report that curcumin (CUR), a naturally occurring compound endowed with p300/CREB-binding protein HAT inhibitory activity, is able to induce a drastic down-regulation of the mGlu2 receptor in the mouse spinal cord after systemic administration together with a marked hypoacetylation of histones H3 and H4 in dorsal root ganglia (DRG). Furthermore, the analgesic activity of the mGlu2/3 agonist, LY379268 is lost after a 3-day treatment with CUR. Conversely the analgesic activity of LY379268 is potentiated in mice pretreated for 5 consecutive days with the HDAC inhibitor, Suberoylanilide Hydroxamic Acid (SAHA), known to induce mGlu2-upregulation.

**Conclusions:**

Our results demonstrate that systemically injected CUR is able to inhibit H3 and H4 acetylation in the DRG and to down-regulate mGlu2 receptors in the spinal cord. We also demonstrate that long term modification of the mGlu2 expression affects the analgesic properties of the orthosteric mGlu2/3 agonist, LY379268. These data open up the possibility that epigenetic modulators might be given in combination with “traditional” drugs in a context of a multi target approach for a better analgesic efficacy.

## Findings

### Background

Histone deacetylases (HDACs) and histone acetyltransferases (HATs) are two families of enzymes that regulate the acetylation status of lysine residues of histone tails, thus behaving as a chromatin modulators. The hyper- or hypo-acetylation state of chromatin impacts on the accessibility of specific DNA sequences to transcription factors and other modulators of gene expression. Thus, HDAC or HAT inhibitors act as epigenetic modulators able to induce long-term changes in gene expression [1, 2]. Beside histones, HDACs and HATs also target non-histone proteins and transcription factors therefore modulating the expression of a number of receptors, ion channels and other downstream targets [3]. HDAC inhibitors and acetylating agents have been shown to induce analgesia in different models of inflammatory pain [1, 4–9] and to alleviate nerve injury-induced hypersensitivity [8–14]. Several epigenetic mechanisms have been proposed to explain the analgesic activity of HDAC inhibitors. We first reported that an increased expression of the metabotropic glutamate receptor type 2 (mGlu2) in the spinal cord and dorsal root ganglia (DRG) is responsible for the analgesic effect of two different HDAC inhibitors such as suberoylanilide hydroxamic acid (SAHA) and *N*-(2-aminophenyl)-4-[*N*-(pyridine-3-ylmethoxy-carbonyl)aminomethyl] benzamide (MS275) in the second phase of the formalin test [5]. The mechanism underlying the mGlu2 overexpression induced by HDAC inhibitors has been related to the activation of the NF-κB pathway by an increased acetylation of the p65 subunit at lysine 310 (K310) [5, 6].

With this study we sought to evaluate whether inhibition of HAT activity might affect mGlu2 expression and thus have an impact on mGlu2-mediated analgesia. To test this hypothesis we used a naturally occurring compound endowed with p300/CREB-binding protein (CBP) histone HAT inhibitory activity, curcumin (CUR), extracted from rhizomes of turmeric Curcuma longa [15–17]. We also evaluated the analgesic effect of the mGlu2/3 agonist, LY379268, in mice in which the expression of the mGlu2 receptor was epigenetically modulated.

## Results and discussion

**Figure 1:**
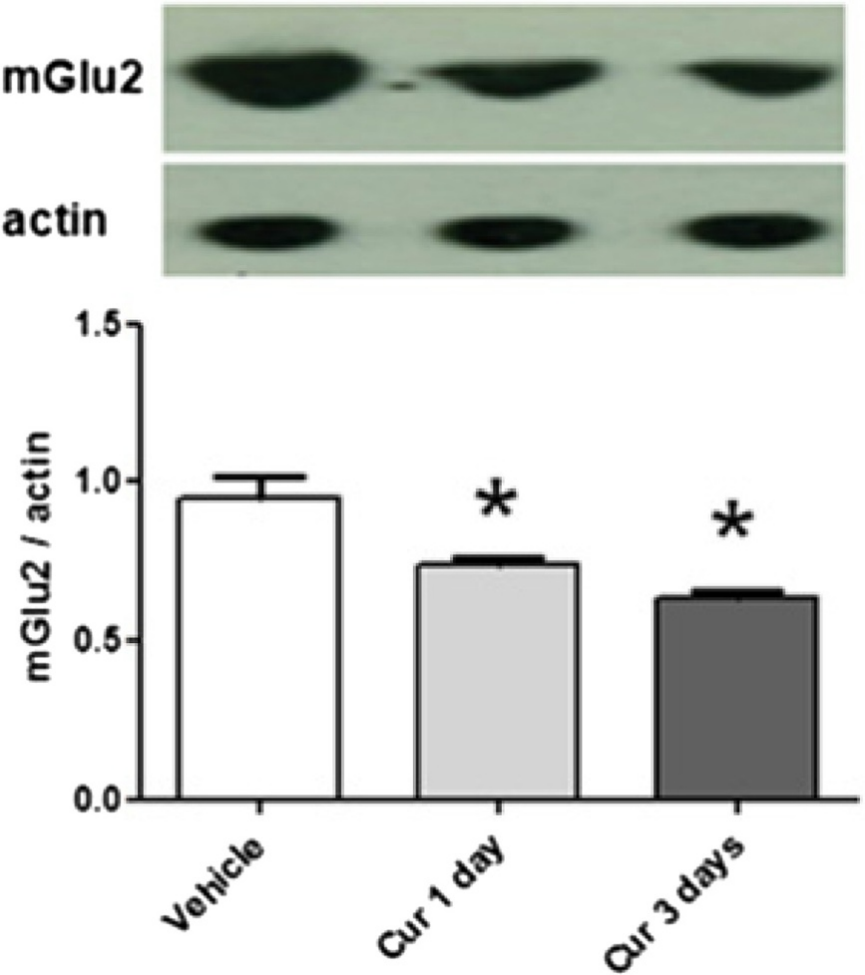
**Expression of mGlu2 receptors in the mouse spinal cord after curcumin treatment.** A single or repeated injections of CUR (100 mg/kg, ip) decreased the expression of mGlu2 receptors in the lumbar segment of the spinal cord. Data are the means ± S.E.M. of 4 animals. *p < 0.05 (Student’s *t* test) versus values obtained in animals treated with vehicle.

**Figure 2:**
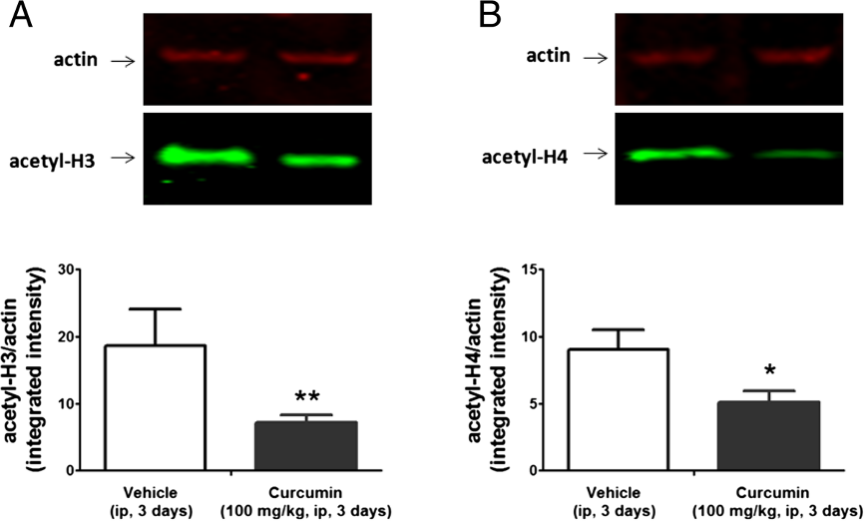
**Expression of acetyl-H3 and acetyl-H4 in the mouse dorsal root ganglia after a 3-day curcumin pretreatment.** Repeated injection of curcumin (100 mg/kg, ip, for three days) reduced the expression of acetyl-H3 and acetyl-H4 in the DRG. DRGs were dissected on the third day, 24 hours after the last administration. A representative immunoblot of acetylated-H3 and acetylated-H4 in DRG extracts from mice treated with curcumin is shown in **(A)** and **(B)** respectively. Densitometric analysis of acetyl-H3 and acetyl-H4 normalized by actin is shown. Data are the means ± S.E.M. of 4 animals. *p < 0.05 (Student’s *t* test) versus values obtained in animals treated with vehicle.

**Figure 3:**
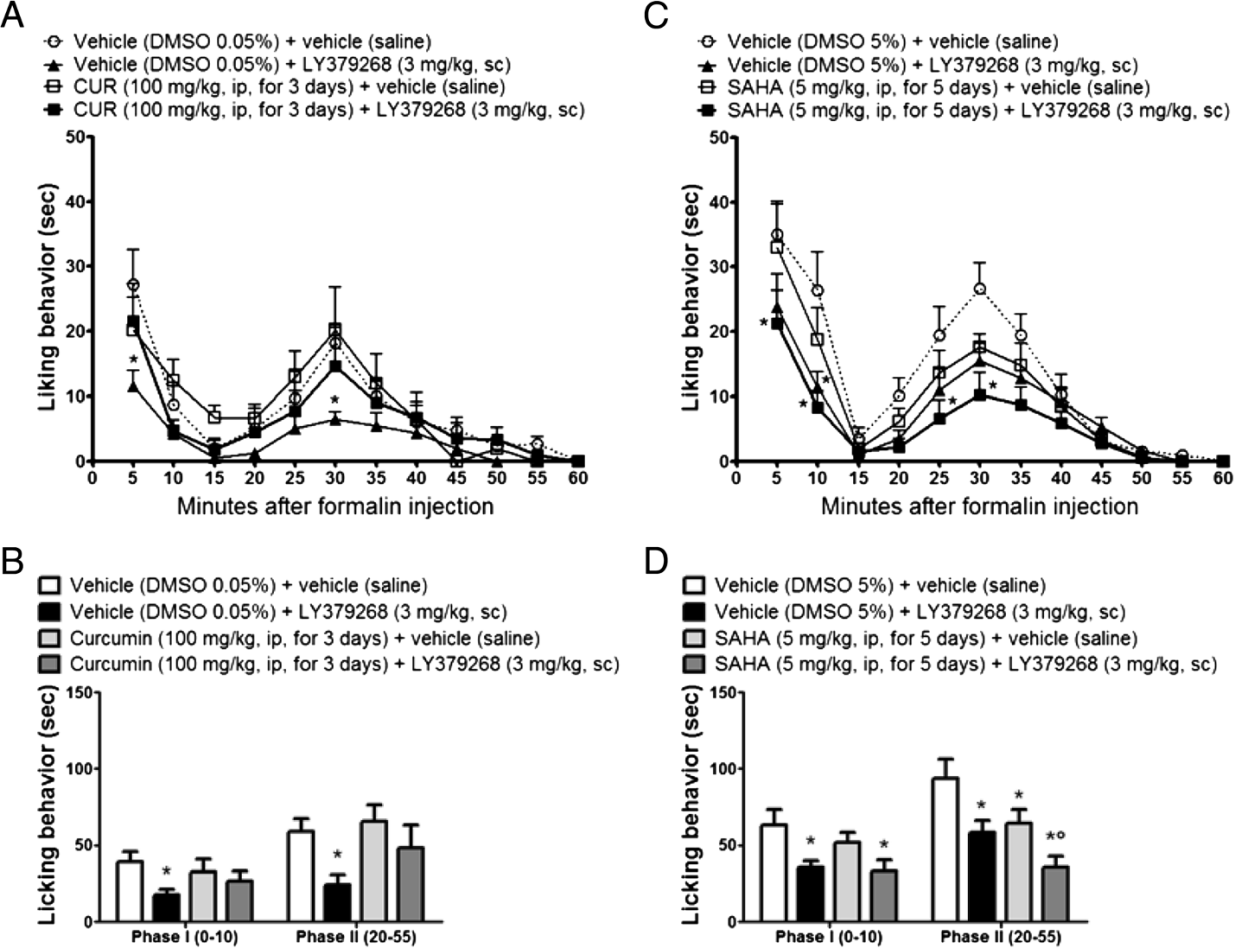
**CUR and SAHA differently affect the analgesic efficacy of LY379268 in the mouse formalin test. (A, B)** CUR-treated mice (100 mg/kg, ip for three consecutive days) did not significantly differ from vehicle-treated mice. The acute administration of LY379268 (3 mg/kg, i.p.) 30 minutes before formalin injection significantly reduced both phases in mice. A single administration of LY379268 (3 mg/kg, i.p.) 30 min before formalin in CUR-pretreated mice failed to induce analgesia in both phases of the formalin test. **(C, D)** SAHA treated mice (5 mg/kg, sc, for 5 consecutive days) significantly reduced the licking behavior in the second phase of the formalin test. A single administration of LY379268 (3 mg/kg, i.p.) 30 minutes before formalin injection significantly reduced both phases in mice. The analgesic effect of LY378268 acutely injected 30 min before formalin was potentiated in SAHA-pretreated mice. Data represent the mean ± S.E.M. of 12 to 16 mice per group. *p < 0.05 (Two-way ANOVA + Bonferroni) versus the respective vehicle group, °*p* < 0.05 (Two-way ANOVA + Bonferroni) versus the corresponding group treated with SAHA.

We examined the effect of repeated injection with the naturally occurring p300/CBP HAT inhibitor, CUR, on the mGlu2 expression level in the spinal cord. Chronic administration of CUR at doses ranging from 50 to 300 mg/kg have been shown to be well tolerated in behavioral studies [18]. Intraperitonally injected CUR (100 mg/kg) is able to decrease histone acetylation in the CNS only after inclusion in nanostructured lipid carriers [17]. In this study we demonstrated that CUR (100 mg/kg, ip) was able to significantly reduce the mGlu2 receptor expression in the mouse spinal cord after 24 hours from the injection with a lowest level reached after 3 consecutive days of administration (Figure 1). However no further reduction in mGlu2 receptor expression was observed after a more prolonged treatment such as a 5-day treatment (data not shown). Systemically injected, CUR was also able to induce a significant H3 and H4 hypoacetylation in the DRG (Figure 2A, 2B). Since mGlu2 receptors play an important role in pain behavior and in the analgesic effects of mGlu2/3 agonists [19] we next evaluated whether a 3-day CUR administration has an impact on pain behavior in the mouse formalin test and/or affects the analgesic activity of the mGlu2/3 agonist, LY379268. mGlu2/3 agonists are known to induce analgesia in different pain models including the formalin test [10, 19–29]. Consistent with data from literature, a single administration of LY379268 (3 mg/kg, ip) 30 minutes before formalin injection, was able to induce analgesia in the mouse formalin test (Figure 3A-D). Also, to test the effect of a 3-day treatment with CUR on the analgesic activity of LY379268 we pretreated mice with CUR (100 mg/kg, ip) for three consecutive days with the last injection 24 hours before the formalin test and then mice received either saline or LY379268 30 minute before the formalin injection. Although CUR was able to induce mGlu2 downregulation, mice receiving a 3-day pretreatment with CUR exhibited a slight but not significant increase of pain behavior in the second phase of the formalin test compared to saline-injected mice (Figure 3A,B). However, under these conditions, the analgesic effect of LY379268, was lost (Figure 3A,B). We then moved to test whether repeated injection with the HDAC inhibitor, SAHA, would affect the analgesic activity of the mGlu2/3 agonist, LY379268. To address this question we pretreated mice with SAHA (5 mg/Kg, sc) for 5 consecutive days. As previously shown [5], while a single injection of SAHA is not analgesic and does not affect mGlu2 receptor expression in the mouse spinal cord, a 5-day pretreatment is sufficient to upregulate mGlu2 receptors and to reduce the second phase of the mouse formalin test [5]. Here we show that, opposed to Curcumin, when mice were pretreated with SAHA (5 mg/Kg, sc) for 5 consecutive days with the last injection 24 hours before the formalin injection the analgesic effect of LY379268 (3 mg/kg, ip), acutely injected 30 minutes before formalin injection, was potentiated in the second phase of the formalin test (Figure 3C,D).

Our data show that epigenetic modulation of mGlu2 receptors affects the analgesic activity of orthosteric mGlu2/3 ligands. It has been shown that mGlu2 expression is regulated by epigenetic mechanism in peripheral and central nervous system regions including DRG, spinal cord [5, 9], and prefrontal cortex [30]. Particularly, pharmacological treatments leading to protein hyperacetylation, such as HDAC inhibitors or L-acetylcarnitine, have been shown to increase the expression of mGlu2 receptor both in *in vivo* and *in vitro* experiments [5, 6, 9, 10, 30–32] and to induce analgesia in the second phase of the mouse formalin test [5, 6]. However, no report indicates whether a decreased protein acetylation level might influence the expression of mGlu2 receptor and thus have an impact on mGlu2/3 agonist-induced analgesia. As a p300/CBP HAT inhibitor, CUR can contribute to the regulation of gene expression [15, 16]. p300/CBP is ubiquitously expressed and plays an important role in a wide range of biological responses involved in inflammation, cancer and neurodegenerative diseases [26, 33, 34]. Although CUR-induced p300/CBP inhibition results in a consistent mGlu2 receptor downregulation, the lack of hyperalgesic activity of CUR might be related to the lack of specificity of HAT inhibitors. A number of targets have been shown to be modulated by CUR, many of which might impact pain behavior [27]. Based on our previous works showing that HDAC inhibitor regulate mGlu2 receptor expression via NF-κB activation [5, 6], here we focus on the ability of CUR to epigenetically downregulate mGlu2 receptor in DRG and thus to modulate mGlu2/3 analgesic activity. Interestingly, CUR also inhibits NF-κB activation and the expression of its target genes [35]. We have previously shown that systemic administration of CUR is not able to induce histone hypoacetylation in the spinal cord unless CUR is included in a nanocarrier lipid matrix [17]. This is consistent with the low bioavailability of CUR because of its rapid metabolism and pharmacokinetic characteristics that do not allow the drug to reach high concentration in the CNS [36]. In our study we show that systemically injected CUR is able to induce H3 and H4 hypoacetylation in the DRG. Although CUR is not able to induce hypoacetylation in the spinal cord after systemic injection, the reduced acetylation observed in the DRG might be explained by the lack of the blood brain barrier that increases DRG neuron vulnerability compared to the central nervous system. The effect of CUR in the DRG also explains the downregulation of mGlu2 receptors in the spinal cord. In fact, mGlu2 receptors in the dorsal horn of the spinal cord are presynaptically expressed in the primary sensory afferents whose cell bodies are located in the DRG [28]. the presynaptic localization of mGu2 receptors on primary afferent neurons, together with the absence of blood brain barrier in DRG also explain the effect of SAHA on mGlu2 receptor expression in the spinal cord despite its poor brain permeability [29]. The lack of analgesic effect in the formalin test by the mGlu2/3 agonist LY379268 in CUR-treated mice is consistent with our previous work showing that LY379268 is not analgesic in mice lacking of the mGlu2 receptors [19, 37]. Recently, the role of p300 in chronic pain has been investigated. Zhu and colleagues [38] show that p300 is involved in the development of neuropathic pain and that a reversal of hyperalgesia can be induced by strategies that inhibit or down-regulate p300 in the spinal cord. The authors showed that p300 is up-regulated at day 14 after the induction of neuropathic pain with the chronic constriction injury of the sciatic nerve model. Moreover, the inhibition of p300 HAT by intrathecal administration of a small molecule with p300 HAT inhibitory activity, C646, or by down-regulating p300 with specific small hairpin RNA (shRNA) suppress cyclooxygenase-2 (COX-2) expression [38]. These data are not in contrast with our result showing that the p300/CBP HAT inhibitor, CUR, does not affect pain behavior in the formalin test, since we have shown that, when systemically injected, CUR affects histone acetylation in the DRG, but not in the spinal cord [17]. Consistent with this hypothesis, CUR has been shown to reduce the second phase of the formalin test when intrathecally injected [39].

In this short report we show that a bidirectional modulation of mGlu2 expression in the spinal cord induced by repeated administration of so called “epigenetic drugs”, such as HDACs or HATs inhibitors, can either potentiate or block the analgesic activity of the mGlu2/3 agonist, LY379268.

## Methods

### Animals

Adult (8–10 weeks) Wild type CD1 mice Were used in this study. All mice were housed under a 12 h light/dark cycle with free access to food and water. All mouse protocols are in accordance with Institutional Animal Care and Use Committee (IACUC) guidelines. All efforts were made to minimize animal suffering and to reduce the number of animals used.

### Drugs

Curcumin (Sigma-Aldrich) was dissolved in normal saline solution containing 0.05% dimethyl sulfoxide (DMSO), as previously described [17]. Curcumin was administered to CD1 mice at 100 mg/kg body weight intraperitoneally (i.p.) once a day for three consecutive days with the last injection 24 h before behavioral test. A vehicle solution containing 0.05% DMSO was given as control for the curcumin group. SAHA (Calbiochem) was dissolved in 5% DMSO and injected at 5 mg/kg subcutaneously for 5 consecutive days with the last injection 24 h before behavioral tests. LY379268 (Toscris) was acutely injected at 3 mg/Kg, sc 30 min before the formalin test. A vehicle solution containing 5% DMSO was given as control for the SAHA group.

### Behavioral experiments

#### Formalin test

Formalin (5%, 10 μl; Sigma-Aldrich) was injected subcutaneously into the plantar side of the right hind paw as reported previously [5]. In the formalin test, the total time spent licking or biting the injected hind paw was recorded for each five-minute intervals for one hour post injection. Formalin scores were separated into two phases, phase I (0–10 min) and phase II (15–50 min). A mean response was then calculated for each phase. All tests were performed blind to treatment.

### Immunoblotting

Tissues from the lumbar segment of the spinal cord or DRG from L4–L5 were removed and homogenized. Ten micrograms of total protein were separated by 10% SDS polyacrylamide gel electrophoresis and electrophoretically transferred onto protein-sensitive nitrocellulose membranes (Criterion blotter; Bio-Rad Laboratories, Hercules, CA). The membranes were blocked in Odyssey blocker (LI-COR Biosciences, Lincoln, NE) for 1 h, and the following primary antibodies were used: rabbit anti-acetyl H3 (1:1000; Santa Cruz Biotechnology, Santa Cruz, CA); rabbit Ac-Histone H4 (Lys 12) (1:200, Santa Cruz Biotechnology, Santa Cruz, CA); rabbit Ac-Histone H3 (Lys 18) (1:200, Abcam); mouse anti actin (1:1000; Sigma Aldrich). Mouse anti-metabotropic Glutamate Receptor 2 (1:500; Abcam). Primary antibodies were incubated overnight at 4°C. The following secondary antibodies were incubated for 1 h at room temperature: goat anti-rabbit antibody labeled with IRD800CW (LICOR) and goat anti-mouse antibody labeled with Alexa Fluor 680 (Invitrogen, Carlsbad, CA). Proteins were detected with the Odyssey Infrared Fluorescence Imaging System (LI-COR).
